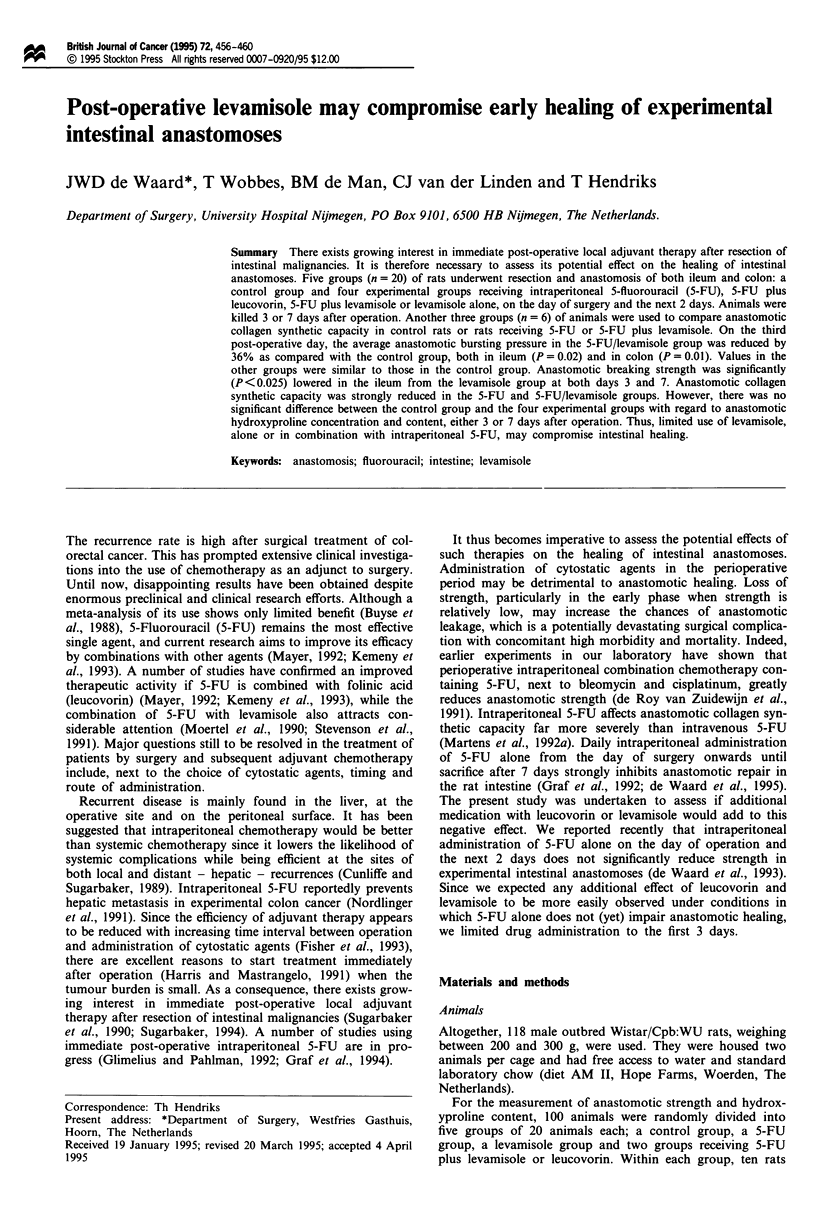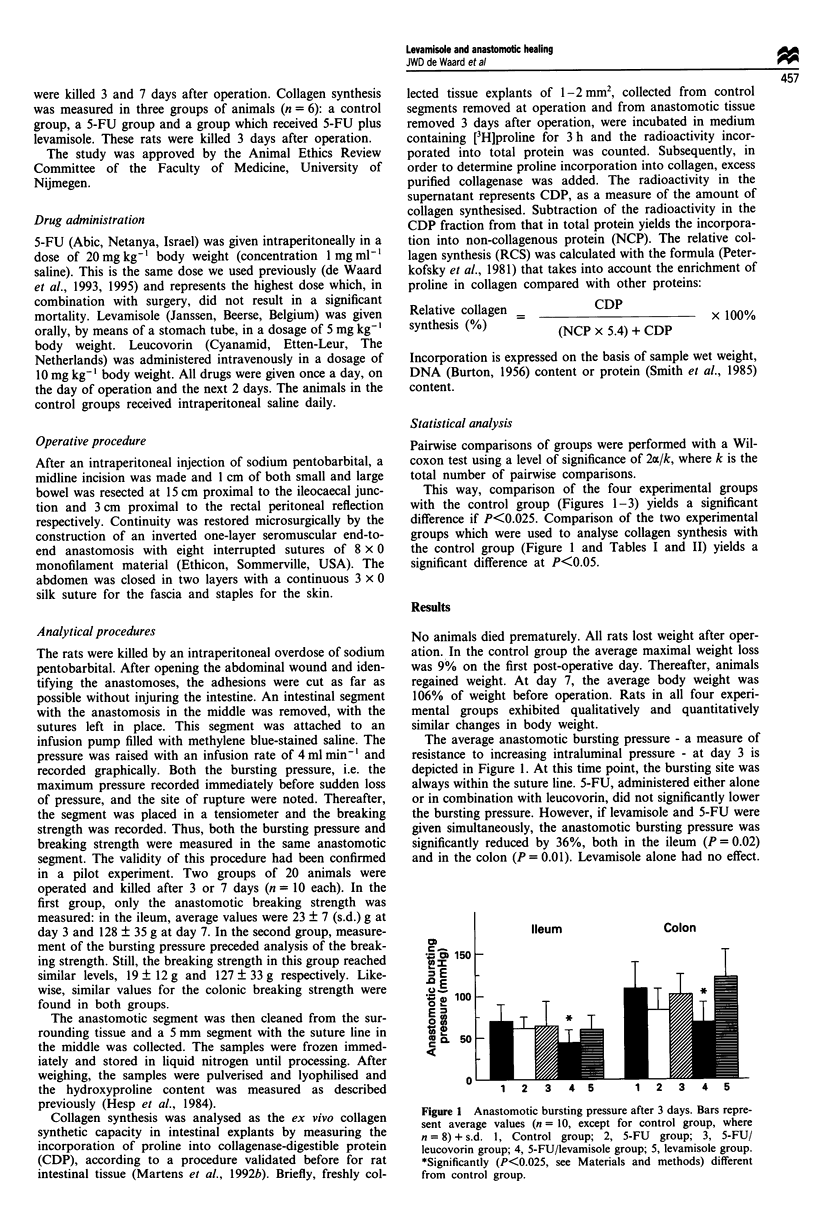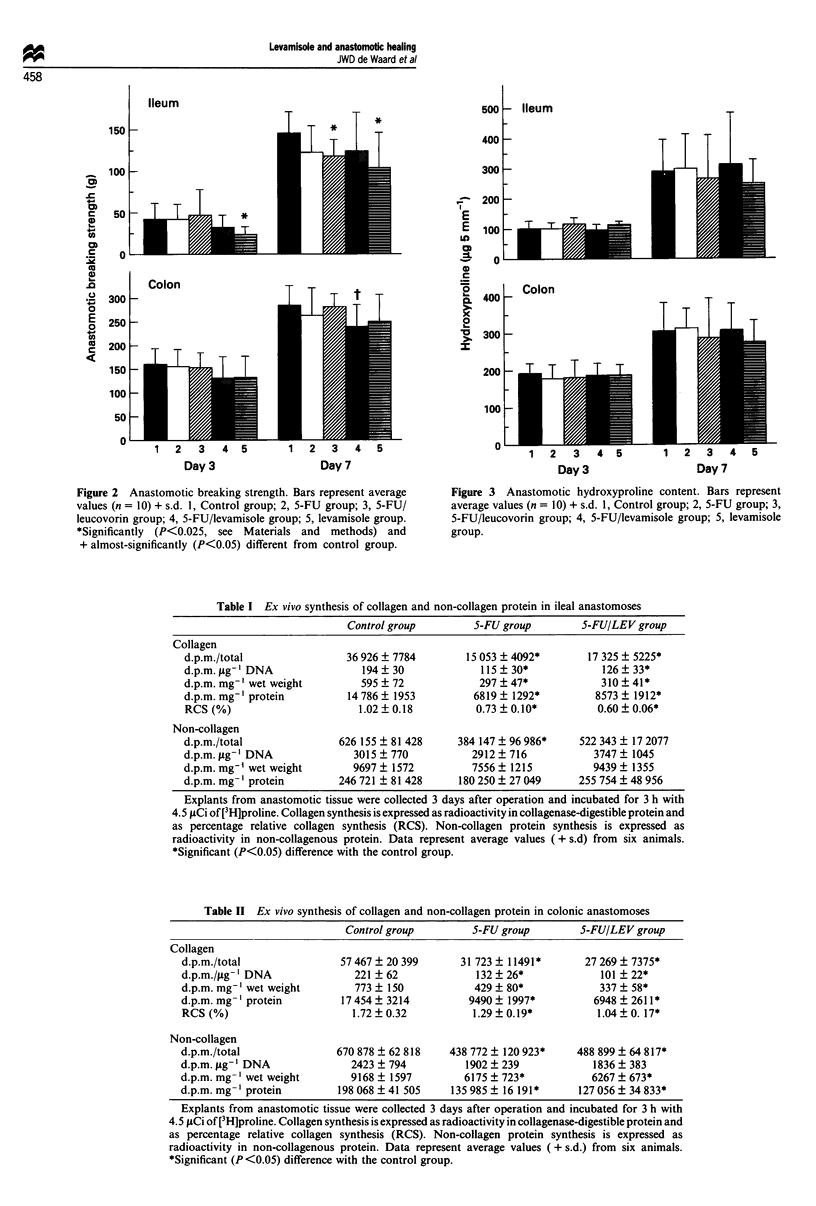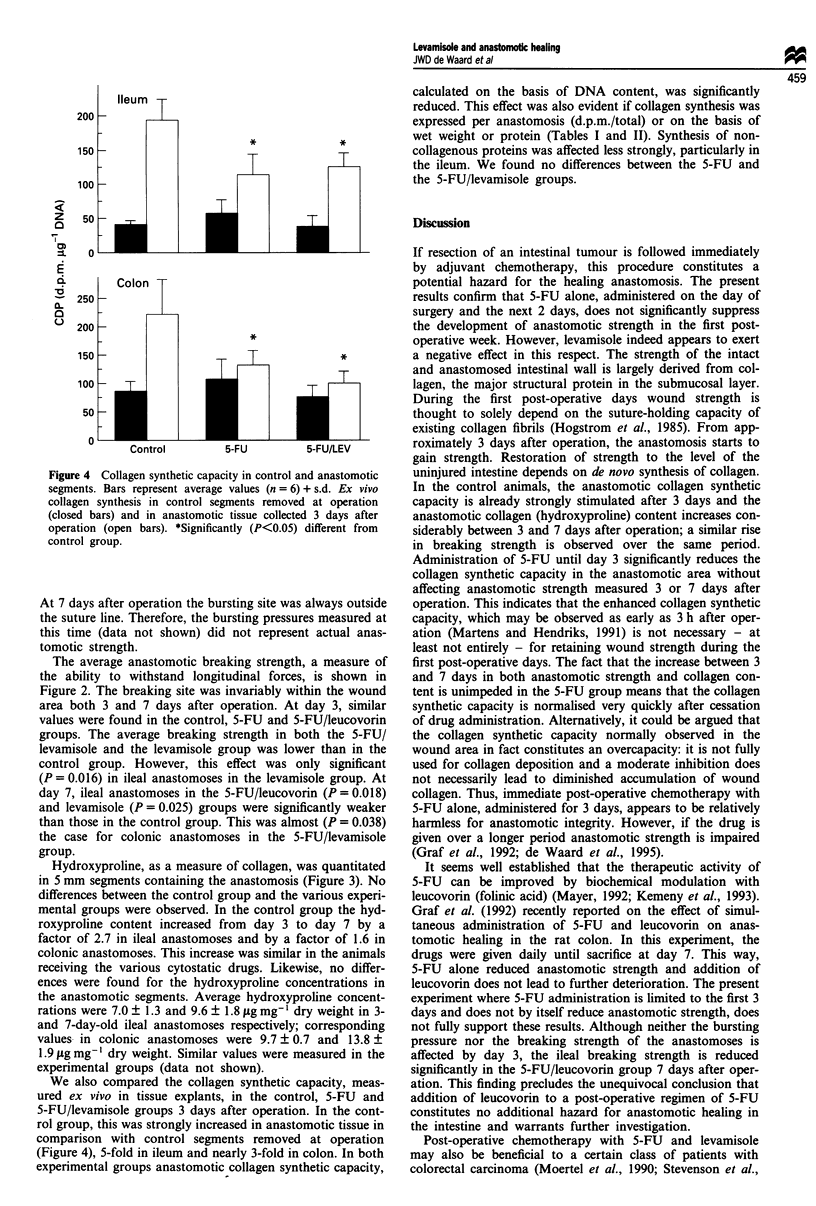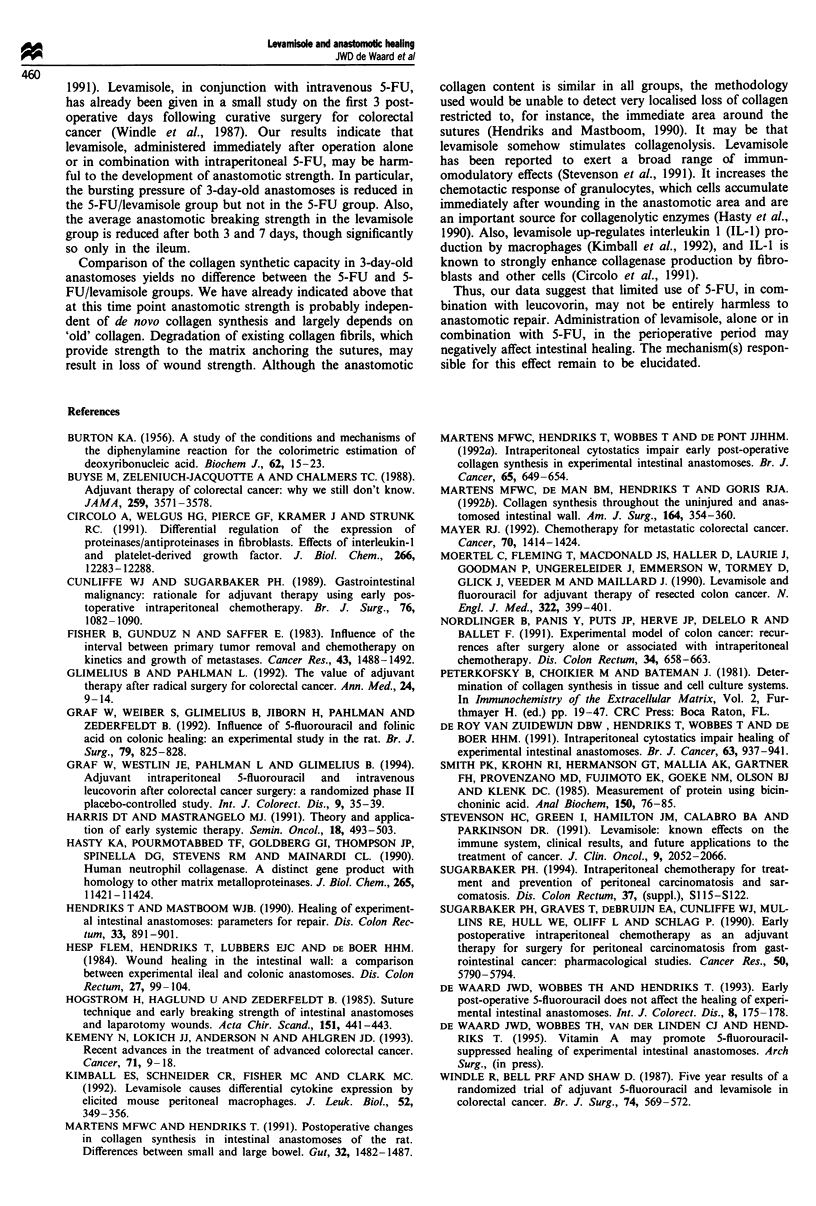# Post-operative levamisole may compromise early healing of experimental intestinal anastomoses.

**DOI:** 10.1038/bjc.1995.355

**Published:** 1995-08

**Authors:** J. W. de Waard, T. Wobbes, B. M. de Man, C. J. van der Linden, T. Hendriks

**Affiliations:** Department of Surgery, University Hospital Nijmegen, The Netherlands.

## Abstract

There exists growing interest in immediate post-operative local adjuvant therapy after resection of intestinal malignancies. It is therefore necessary to assess it potential effect on the healing of intestinal anastomoses. Five groups (n = 20) of rats underwent resection and anastomosis of both ileum and colon: a control group and four experimental groups receiving intraperitoneal 5-fluorouracil (5-FU), 5-FU plus leucovorin, 5-FU plus levamisole or levamisole alone, on the day of surgery and the next 2 days. Animals were killed 3 or 7 days after operation. Another three groups (n = 6) of animals were used to compare anastomotic collagen synthetic capacity in control rats or rats receiving 5-FU or 5-FU plus levamisole. On the third post-operative day, the average anastomotic bursting pressure in the 5-FU/levamisole group was reduced by 36% as compared with the control group, both in ileum (P = 0.02) and in colon (P = 0.01). Values in the other groups were similar to those in the control group. Anastomotic breaking strength was significantly (P < 0.025) lowered in the ileum from the levamisole group at both days 3 and 7. Anastomotic collagen synthetic capacity was strongly reduced in the 5-FU and 5-FU/levamisole groups. However, there was no significant difference between the control group and the four experimental groups with regard to anastomotic hydroxyproline concentration and content, either 3 or 7 days after operation. Thus, limited use of levamisole, alone or in combination with intraperitoneal 5-FU, may compromise intestinal healing.


British Journal of Cancer (1995) 72, 456-460
© 1995 Stockton Press All rights reserved 0007-0920/95 $12.00

### Post-operative levamisole may compromise early healing of experimental intestinal anastomoses

JWD de Waard*, T Wobbes, BM de Man, CJ van der Linden and T Hendriks

*Department of Surgery, University Hospital Nijmegen, PO Box 9101, 6500 HB Nijmegen, The Netherlands.*

**Summary** There exists growing interest in immediate post-operative local adjuvant therapy after resection of intestinal malignancies. It is therefore necessary to assess its potential effect on the healing of intestinal anastomoses. Five groups ($n = 20$) of rats underwent resection and anastomosis of both ileum and colon: a control group and four experimental groups receiving intraperitoneal 5-fluorouracil (5-FU), 5-FU plus leucovorin, 5-FU plus levamisole or levamisole alone, on the day of surgery and the next 2 days. Animals were killed 3 or 7 days after operation. Another three groups ($n = 6$) of animals were used to compare anastomotic collagen synthetic capacity in control rats or rats receiving 5-FU or 5-FU plus levamisole. On the third post-operative day, the average anastomotic bursting pressure in the 5-FU/levamisole group was reduced by 36% as compared with the control group, both in ileum ($P = 0.02$) and in colon ($P = 0.01$). Values in the other groups were similar to those in the control group. Anastomotic breaking strength was significantly ($P < 0.025$) lowered in the ileum from the levamisole group at both days 3 and 7. Anastomotic collagen synthetic capacity was strongly reduced in the 5-FU and 5-FU/levamisole groups. However, there was no significant difference between the control group and the four experimental groups with regard to anastomotic hydroxyproline concentration and content, either 3 or 7 days after operation. Thus, limited use of levamisole, alone or in combination with intraperitoneal 5-FU, may compromise intestinal healing.

**Keywords:** anastomosis; fluorouracil; intestine; levamisole

The recurrence rate is high after surgical treatment of colorectal cancer. This has prompted extensive clinical investigations into the use of chemotherapy as an adjunct to surgery. Until now, disappointing results have been obtained despite enormous preclinical and clinical research efforts. Although a meta-analysis of its use shows only limited benefit (Buyse *et al.*, 1988), 5-Fluorouracil (5-FU) remains the most effective single agent, and current research aims to improve its efficacy by combinations with other agents (Mayer, 1992; Kemeny *et al.*, 1993). A number of studies have confirmed an improved therapeutic activity if 5-FU is combined with folinic acid (leucovorin) (Mayer, 1992; Kemeny *et al.*, 1993), while the combination of 5-FU with levamisole also attracts considerable attention (Moertel *et al.*, 1990; Stevenson *et al.*, 1991). Major questions still to be resolved in the treatment of patients by surgery and subsequent adjuvant chemotherapy include, next to the choice of cytostatic agents, timing and route of administration.

Recurrent disease is mainly found in the liver, at the operative site and on the peritoneal surface. It has been suggested that intraperitoneal chemotherapy would be better than systemic chemotherapy since it lowers the likelihood of systemic complications while being efficient at the sites of both local and distant – hepatic – recurrences (Cunliffe and Sugarbaker, 1989). Intraperitoneal 5-FU reportedly prevents hepatic metastasis in experimental colon cancer (Nordlinger *et al.*, 1991). Since the efficiency of adjuvant therapy appears to be reduced with increasing time interval between operation and administration of cytostatic agents (Fisher *et al.*, 1993), there are excellent reasons to start treatment immediately after operation (Harris and Mastrangelo, 1991) when the tumour burden is small. As a consequence, there exists growing interest in immediate post-operative local adjuvant therapy after resection of intestinal malignancies (Sugarbaker *et al.*, 1990; Sugarbaker, 1994). A number of studies using immediate post-operative intraperitoneal 5-FU are in progress (Glimelius and Pahlman, 1992; Graf *et al.*, 1994).

Correspondence: Th Hendriks
Present address: *Department of Surgery, Westfries Gasthuis, Hoorn, The Netherlands
Received 19 January 1995; revised 20 March 1995; accepted 4 April 1995

It thus becomes imperative to assess the potential effects of such therapies on the healing of intestinal anastomoses. Administration of cytostatic agents in the perioperative period may be detrimental to anastomotic healing. Loss of strength, particularly in the early phase when strength is relatively low, may increase the chances of anastomotic leakage, which is a potentially devastating surgical complication with concomitant high morbidity and mortality. Indeed, earlier experiments in our laboratory have shown that perioperative intraperitoneal combination chemotherapy containing 5-FU, next to bleomycin and cisplatinum, greatly reduces anastomotic strength (de Roy van Zuidewijn *et al.*, 1991). Intraperitoneal 5-FU affects anastomotic collagen synthetic capacity far more severely than intravenous 5-FU (Martens *et al.*, 1992*a*). Daily intraperitoneal administration of 5-FU alone from the day of surgery onwards until sacrifice after 7 days strongly inhibits anastomotic repair in the rat intestine (Graf *et al.*, 1992; de Waard *et al.*, 1995). The present study was undertaken to assess if additional medication with leucovorin or levamisole would add to this negative effect. We reported recently that intraperitoneal administration of 5-FU alone on the day of operation and the next 2 days does not significantly reduce strength in experimental intestinal anastomoses (de Waard *et al.*, 1993). Since we expected any additional effect of leucovorin and levamisole to be more easily observed under conditions in which 5-FU alone does not (yet) impair anastomotic healing, we limited drug administration to the first 3 days.

### Materials and methods

### *Animals*

Altogether, 118 male outbred Wistar/Cpb:WU rats, weighing between 200 and 300 g, were used. They were housed two animals per cage and had free access to water and standard laboratory chow (diet AM II, Hope Farms, Woerden, The Netherlands).

For the measurement of anastomotic strength and hydroxyproline content, 100 animals were randomly divided into five groups of 20 animals each; a control group, a 5-FU group, a levamisole group and two groups receiving 5-FU plus levamisole or leucovorin. Within each group, ten rats were killed 3 and 7 days after operation. Collagen synthesis was measured in three groups of animals ($n = 6$): a control group, a 5-FU group and a group which received 5-FU plus levamisole. These rats were killed 3 days after operation.

The study was approved by the Animal Ethics Review Committee of the Faculty of Medicine, University of Nijmegen.

### *Drug administration*

5-FU (Abic, Netanya, Israel) was given intraperitoneally in a dose of 20 mg $kg^{-1}$ body weight (concentration 1 mg $ml^{-1}$ saline). This is the same dose we used previously (de Waard *et al.*, 1993, 1995) and represents the highest dose which, in combination with surgery, did not result in a significant mortality. Levamisole (Janssen, Beerse, Belgium) was given orally, by means of a stomach tube, in a dosage of 5 mg $kg^{-1}$ body weight. Leucovorin (Cyanamid, Etten-Leur, The Netherlands) was administered intravenously in a dosage of 10 mg $kg^{-1}$ body weight. All drugs were given once a day, on the day of operation and the next 2 days. The animals in the control groups received intraperitoneal saline daily.

### *Operative procedure*

After an intraperitoneal injection of sodium pentobarbital, a midline incision was made and 1 cm of both small and large bowel was resected at 15 cm proximal to the ileocaecal junction and 3 cm proximal to the rectal peritoneal reflection respectively. Continuity was restored microsurgically by the construction of an inverted one-layer seromuscular end-to-end anastomosis with eight interrupted sutures of 8 × 0 monofilament material (Ethicon, Sommerville, USA). The abdomen was closed in two layers with a continuous 3 × 0 silk suture for the fascia and staples for the skin.

### *Analytical procedures*

The rats were killed by an intraperitoneal overdose of sodium pentobarbital. After opening the abdominal wound and identifying the anastomoses, the adhesions were cut as far as possible without injuring the intestine. An intestinal segment with the anastomosis in the middle was removed, with the sutures left in place. This segment was attached to an infusion pump filled with methylene blue-stained saline. The pressure was raised with an infusion rate of 4 ml $min^{-1}$ and recorded graphically. Both the bursting pressure, i.e. the maximum pressure recorded immediately before sudden loss of pressure, and the site of rupture were noted. Thereafter, the segment was placed in a tensiometer and the breaking strength was recorded. Thus, both the bursting pressure and breaking strength were measured in the same anastomotic segment. The validity of this procedure had been confirmed in a pilot experiment. Two groups of 20 animals were operated and killed after 3 or 7 days ($n = 10$ each). In the first group, only the anastomotic breaking strength was measured: in the ileum, average values were 23 ± 7 (s.d.) g at day 3 and 128 ± 35 g at day 7. In the second group, measurement of the bursting pressure preceded analysis of the breaking strength. Still, the breaking strength in this group reached similar levels, 19 ± 12 g and 127 ± 33 g respectively. Likewise, similar values for the colonic breaking strength were found in both groups.

The anastomotic segment was then cleaned from the surrounding tissue and a 5 mm segment with the suture line in the middle was collected. The samples were frozen immediately and stored in liquid nitrogen until processing. After weighing, the samples were pulverised and lyophilised and the hydroxyproline content was measured as described previously (Hesp *et al.*, 1984).

Collagen synthesis was analysed as the *ex vivo* collagen synthetic capacity in intestinal explants by measuring the incorporation of proline into collagenase-digestible protein (CDP), according to a procedure validated before for rat intestinal tissue (Martens *et al.*, 1992*b*). Briefly, freshly collected tissue explants of 1–2 $mm^2$, collected from control segments removed at operation and from anastomotic tissue removed 3 days after operation, were incubated in medium containing [$^3$H]proline for 3 h and the radioactivity incorporated into total protein was counted. Subsequently, in order to determine proline incorporation into collagen, excess purified collagenase was added. The radioactivity in the supernatant represents CDP, as a measure of the amount of collagen synthesised. Subtraction of the radioactivity in the CDP fraction from that in total protein yields the incorporation into non-collagenous protein (NCP). The relative collagen synthesis (RCS) was calculated with the formula (Peterkofsky *et al.*, 1981) that takes into account the enrichment of proline in collagen compared with other proteins:

$$\text{Relative collagen synthesis (\%)} = \frac{\text{CDP}}{(\text{NCP} \times 5.4) + \text{CDP}} \times 100\%$$

Incorporation is expressed on the basis of sample wet weight, DNA (Burton, 1956) content or protein (Smith *et al.*, 1985) content.

### *Statistical analysis*

Pairwise comparisons of groups were performed with a Wilcoxon test using a level of significance of $2\alpha/k$, where $k$ is the total number of pairwise comparisons.

This way, comparison of the four experimental groups with the control group (Figures 1–3) yields a significant difference if $P<0.025$. Comparison of the two experimental groups which were used to analyse collagen synthesis with the control group (Figure 1 and Tables I and II) yields a significant difference at $P<0.05$.

### Results

No animals died prematurely. All rats lost weight after operation. In the control group the average maximal weight loss was 9% on the first post-operative day. Thereafter, animals regained weight. At day 7, the average body weight was 106% of weight before operation. Rats in all four experimental groups exhibited qualitatively and quantitatively similar changes in body weight.

The average anastomotic bursting pressure - a measure of resistance to increasing intraluminal pressure - at day 3 is depicted in Figure 1. At this time point, the bursting site was always within the suture line. 5-FU, administered either alone or in combination with leucovorin, did not significantly lower the bursting pressure. However, if levamisole and 5-FU were given simultaneously, the anastomotic bursting pressure was significantly reduced by 36%, both in the ileum ($P = 0.02$) and in the colon ($P = 0.01$). Levamisole alone had no effect.

**Figure 1** Anastomotic bursting pressure after 3 days. Bars represent average values ($n = 10$, except for control group, where $n = 8$) + s.d. 1, Control group; 2, 5-FU group; 3, 5-FU/leucovorin group; 4, 5-FU/levamisole group; 5, levamisole group. *Significantly ($P<0.025$, see Materials and methods) different from control group.

**Figure 2** Anastomotic breaking strength. Bars represent average values ($n = 10$) + s.d. 1, Control group; 2, 5-FU group; 3, 5-FU/leucovorin group; 4, 5-FU/levamisole group; 5, levamisole group. *Significantly ($P<0.025$, see Materials and methods) and + almost-significantly ($P<0.05$) different from control group.

**Figure 3** Anastomotic hydroxyproline content. Bars represent average values ($n = 10$) + s.d. 1, Control group; 2, 5-FU group; 3, 5-FU/leucovorin group; 4, 5-FU/levamisole group; 5, levamisole group.

**Table I** *Ex vivo* synthesis of collagen and non-collagen protein in ileal anastomoses

| | *Control group* | *5-FU group* | *5-FU/LEV group* |
|---|---|---|---|
| Collagen | | | |
| d.p.m./total | 36 926 ± 7784 | 15 053 ± 4092* | 17 325 ± 5225* |
| d.p.m. $\mu g^{-1}$ DNA | 194 ± 30 | 115 ± 30* | 126 ± 33* |
| d.p.m. $mg^{-1}$ wet weight | 595 ± 72 | 297 ± 47* | 310 ± 41* |
| d.p.m. $mg^{-1}$ protein | 14 786 ± 1953 | 6819 ± 1292* | 8573 ± 1912* |
| RCS (%) | 1.02 ± 0.18 | 0.73 ± 0.10* | 0.60 ± 0.06* |
| Non-collagen | | | |
| d.p.m./total | 626 155 ± 81 428 | 384 147 ± 96 986* | 522 343 ± 17 2077 |
| d.p.m. $\mu g^{-1}$ DNA | 3015 ± 770 | 2912 ± 716 | 3747 ± 1045 |
| d.p.m. $mg^{-1}$ wet weight | 9697 ± 1572 | 7556 ± 1215 | 9439 ± 1355 |
| d.p.m. $mg^{-1}$ protein | 246 721 ± 81 428 | 180 250 ± 27 049 | 255 754 ± 48 956 |

Explants from anastomotic tissue were collected 3 days after operation and incubated for 3 h with 4.5 µCi of [$^3$H]proline. Collagen synthesis is expressed as radioactivity in collagenase-digestible protein and as percentage relative collagen synthesis (RCS). Non-collagen protein synthesis is expressed as radioactivity in non-collagenous protein. Data represent average values ( + s.d) from six animals. *Significant ($P<0.05$) difference with the control group.

**Table II** *Ex vivo* synthesis of collagen and non-collagen protein in colonic anastomoses

| | *Control group* | *5-FU group* | *5-FU/LEV group* |
|---|---|---|---|
| Collagen | | | |
| d.p.m./total | 57 467 ± 20 399 | 31 723 ± 11491* | 27 269 ± 7375* |
| d.p.m./$\mu g^{-1}$ DNA | 221 ± 62 | 132 ± 26* | 101 ± 22* |
| d.p.m. $mg^{-1}$ wet weight | 773 ± 150 | 429 ± 80* | 337 ± 58* |
| d.p.m. $mg^{-1}$ protein | 17 454 ± 3214 | 9490 ± 1997* | 6948 ± 2611* |
| RCS (%) | 1.72 ± 0.32 | 1.29 ± 0.19* | 1.04 ± 0. 17* |
| Non-collagen | | | |
| d.p.m./total | 670 878 ± 62 818 | 438 772 ± 120 923* | 488 899 ± 64 817* |
| d.p.m. $\mu g^{-1}$ DNA | 2423 ± 794 | 1902 ± 239 | 1836 ± 383 |
| d.p.m. $mg^{-1}$ wet weight | 9168 ± 1597 | 6175 ± 723* | 6267 ± 673* |
| d.p.m. $mg^{-1}$ protein | 198 068 ± 41 505 | 135 985 ± 16 191* | 127 056 ± 34 833* |

Explants from anastomotic tissue were collected 3 days after operation and incubated for 3 h with 4.5 µCi of [$^3$H]proline. Collagen synthesis is expressed as radioactivity in collagenase-digestible protein and as percentage relative collagen synthesis (RCS). Non-collagen protein synthesis is expressed as radioactivity in non-collagenous protein. Data represent average values ( + s.d.) from six animals. *Significant ($P<0.05$) difference with the control group.

**Figure 4** Collagen synthetic capacity in control and anastomotic segments. Bars represent average values ($n = 6$) + s.d. *Ex vivo* collagen synthesis in control segments removed at operation (closed bars) and in anastomotic tissue collected 3 days after operation (open bars). *Significantly ($P<0.05$) different from control group.

At 7 days after operation the bursting site was always outside the suture line. Therefore, the bursting pressures measured at this time (data not shown) did not represent actual anastomotic strength.

The average anastomotic breaking strength, a measure of the ability to withstand longitudinal forces, is shown in Figure 2. The breaking site was invariably within the wound area both 3 and 7 days after operation. At day 3, similar values were found in the control, 5-FU and 5-FU/leucovorin groups. The average breaking strength in both the 5-FU/levamisole and the levamisole group was lower than in the control group. However, this effect was only significant ($P = 0.016$) in ileal anastomoses in the levamisole group. At day 7, ileal anastomoses in the 5-FU/leucovorin ($P = 0.018$) and levamisole ($P = 0.025$) groups were significantly weaker than those in the control group. This was almost ($P = 0.038$) the case for colonic anastomoses in the 5-FU/levamisole group.

Hydroxyproline, as a measure of collagen, was quantitated in 5 mm segments containing the anastomosis (Figure 3). No differences between the control group and the various experimental groups were observed. In the control group the hydroxyproline content increased from day 3 to day 7 by a factor of 2.7 in ileal anastomoses and by a factor of 1.6 in colonic anastomoses. This increase was similar in the animals receiving the various cytostatic drugs. Likewise, no differences were found for the hydroxyproline concentrations in the anastomotic segments. Average hydroxyproline concentrations were $7.0 \pm 1.3$ and $9.6 \pm 1.8\ \mu g\ mg^{-1}$ dry weight in 3- and 7-day-old ileal anastomoses respectively; corresponding values in colonic anastomoses were $9.7 \pm 0.7$ and $13.8 \pm 1.9\ \mu g\ mg^{-1}$ dry weight. Similar values were measured in the experimental groups (data not shown).

We also compared the collagen synthetic capacity, measured *ex vivo* in tissue explants, in the control, 5-FU and 5-FU/levamisole groups 3 days after operation. In the control group, this was strongly increased in anastomotic tissue in comparison with control segments removed at operation (Figure 4), 5-fold in ileum and nearly 3-fold in colon. In both experimental groups anastomotic collagen synthetic capacity, calculated on the basis of DNA content, was significantly reduced. This effect was also evident if collagen synthesis was expressed per anastomosis (d.p.m./total) or on the basis of wet weight or protein (Tables I and II). Synthesis of non-collagenous proteins was affected less strongly, particularly in the ileum. We found no differences between the 5-FU and the 5-FU/levamisole groups.

### Discussion

If resection of an intestinal tumour is followed immediately by adjuvant chemotherapy, this procedure constitutes a potential hazard for the healing anastomosis. The present results confirm that 5-FU alone, administered on the day of surgery and the next 2 days, does not significantly suppress the development of anastomotic strength in the first post-operative week. However, levamisole indeed appears to exert a negative effect in this respect. The strength of the intact and anastomosed intestinal wall is largely derived from collagen, the major structural protein in the submucosal layer. During the first post-operative days wound strength is thought to solely depend on the suture-holding capacity of existing collagen fibrils (Hogstrom *et al.*, 1985). From approximately 3 days after operation, the anastomosis starts to gain strength. Restoration of strength to the level of the uninjured intestine depends on *de novo* synthesis of collagen. In the control animals, the anastomotic collagen synthetic capacity is already strongly stimulated after 3 days and the anastomotic collagen (hydroxyproline) content increases considerably between 3 and 7 days after operation; a similar rise in breaking strength is observed over the same period. Administration of 5-FU until day 3 significantly reduces the collagen synthetic capacity in the anastomotic area without affecting anastomotic strength measured 3 or 7 days after operation. This indicates that the enhanced collagen synthetic capacity, which may be observed as early as 3 h after operation (Martens and Hendriks, 1991) is not necessary – at least not entirely – for retaining wound strength during the first post-operative days. The fact that the increase between 3 and 7 days in both anastomotic strength and collagen content is unimpeded in the 5-FU group means that the collagen synthetic capacity is normalised very quickly after cessation of drug administration. Alternatively, it could be argued that the collagen synthetic capacity normally observed in the wound area in fact constitutes an overcapacity: it is not fully used for collagen deposition and a moderate inhibition does not necessarily lead to diminished accumulation of wound collagen. Thus, immediate post-operative chemotherapy with 5-FU alone, administered for 3 days, appears to be relatively harmless for anastomotic integrity. However, if the drug is given over a longer period anastomotic strength is impaired (Graf *et al.*, 1992; de Waard *et al.*, 1995).

It seems well established that the therapeutic activity of 5-FU can be improved by biochemical modulation with leucovorin (folinic acid) (Mayer, 1992; Kemeny *et al.*, 1993). Graf *et al.* (1992) recently reported on the effect of simultaneous administration of 5-FU and leucovorin on anastomotic healing in the rat colon. In this experiment, the drugs were given daily until sacrifice at day 7. This way, 5-FU alone reduced anastomotic strength and addition of leucovorin does not lead to further deterioration. The present experiment where 5-FU administration is limited to the first 3 days and does not by itself reduce anastomotic strength, does not fully support these results. Although neither the bursting pressure nor the breaking strength of the anastomoses is affected by day 3, the ileal breaking strength is reduced significantly in the 5-FU/leucovorin group 7 days after operation. This finding precludes the unequivocal conclusion that addition of leucovorin to a post-operative regimen of 5-FU constitutes no additional hazard for anastomotic healing in the intestine and warrants further investigation.

Post-operative chemotherapy with 5-FU and levamisole may also be beneficial to a certain class of patients with colorectal carcinoma (Moertel *et al.*, 1990; Stevenson *et al.*,

1991). Levamisole, in conjunction with intravenous 5-FU, has already been given in a small study on the first 3 post-operative days following curative surgery for colorectal cancer (Windle *et al.*, 1987). Our results indicate that levamisole, administered immediately after operation alone or in combination with intraperitoneal 5-FU, may be harmful to the development of anastomotic strength. In particular, the bursting pressure of 3-day-old anastomoses is reduced in the 5-FU/levamisole group but not in the 5-FU group. Also, the average anastomotic breaking strength in the levamisole group is reduced after both 3 and 7 days, though significantly so only in the ileum.

Comparison of the collagen synthetic capacity in 3-day-old anastomoses yields no difference between the 5-FU and 5-FU/levamisole groups. We have already indicated above that at this time point anastomotic strength is probably independent of *de novo* collagen synthesis and largely depends on 'old' collagen. Degradation of existing collagen fibrils, which provide strength to the matrix anchoring the sutures, may result in loss of wound strength. Although the anastomotic collagen content is similar in all groups, the methodology used would be unable to detect very localised loss of collagen restricted to, for instance, the immediate area around the sutures (Hendriks and Mastboom, 1990). It may be that levamisole somehow stimulates collagenolysis. Levamisole has been reported to exert a broad range of immunomodulatory effects (Stevenson *et al.*, 1991). It increases the chemotactic response of granulocytes, which cells accumulate immediately after wounding in the anastomotic area and are an important source for collagenolytic enzymes (Hasty *et al.*, 1990). Also, levamisole up-regulates interleukin 1 (IL-1) production by macrophages (Kimball *et al.*, 1992), and IL-1 is known to strongly enhance collagenase production by fibroblasts and other cells (Circolo *et al.*, 1991).

Thus, our data suggest that limited use of 5-FU, in combination with leucovorin, may not be entirely harmless to anastomotic repair. Administration of levamisole, alone or in combination with 5-FU, in the perioperative period may negatively affect intestinal healing. The mechanism(s) responsible for this effect remain to be elucidated.

### References

BURTON KA. (1956). A study of the conditions and mechanisms of the diphenylamine reaction for the colorimetric estimation of deoxyribonucleic acid. *Biochem J.*, **62**, 15–23.

BUYSE M, ZELENIUCH-JACQUOTTE A AND CHALMERS TC. (1988). Adjuvant therapy of colorectal cancer: why we still don't know. *JAMA*, **259**, 3571–3578.

CIRCOLO A, WELGUS HG, PIERCE GF, KRAMER J AND STRUNK RC. (1991). Differential regulation of the expression of proteinases/antiproteinases in fibroblasts. Effects of interleukin-1 and platelet-derived growth factor. *J. Biol. Chem.*, **266**, 12283–12288.

CUNLIFFE WJ AND SUGARBAKER PH. (1989). Gastrointestinal malignancy: rationale for adjuvant therapy using early postoperative intraperitoneal chemotherapy. *Br. J. Surg.*, **76**, 1082–1090.

FISHER B, GUNDUZ N AND SAFFER E. (1983). Influence of the interval between primary tumor removal and chemotherapy on kinetics and growth of metastases. *Cancer Res.*, **43**, 1488–1492.

GLIMELIUS B AND PAHLMAN L. (1992). The value of adjuvant therapy after radical surgery for colorectal cancer. *Ann. Med.*, **24**, 9–14.

GRAF W, WEIBER S, GLIMELIUS B, JIBORN H, PAHLMAN AND ZEDERFELDT B. (1992). Influence of 5-fluorouracil and folinic acid on colonic healing: an experimental study in the rat. *Br. J. Surg.*, **79**, 825–828.

GRAF W, WESTLIN JE, PAHLMAN L AND GLIMELIUS B. (1994). Adjuvant intraperitoneal 5-fluorouracil and intravenous leucovorin after colorectal cancer surgery: a randomized phase II placebo-controlled study. *Int. J. Colorect. Dis.*, **9**, 35–39.

HARRIS DT AND MASTRANGELO MJ. (1991). Theory and application of early systemic therapy. *Semin. Oncol.*, **18**, 493–503.

HASTY KA, POURMOTABBED TF, GOLDBERG GI, THOMPSON JP, SPINELLA DG, STEVENS RM AND MAINARDI CL. (1990). Human neutrophil collagenase. A distinct gene product with homology to other matrix metalloproteinases. *J. Biol. Chem.*, **265**, 11421–11424.

HENDRIKS T AND MASTBOOM WJB. (1990). Healing of experimental intestinal anastomoses: parameters for repair. *Dis. Colon Rectum*, **33**, 891–901.

HESP FLEM, HENDRIKS T, LUBBERS EJC AND DE BOER HHM. (1984). Wound healing in the intestinal wall: a comparison between experimental ileal and colonic anastomoses. *Dis. Colon Rectum*, **27**, 99–104.

HOGSTROM H, HAGLUND U AND ZEDERFELDT B. (1985). Suture technique and early breaking strength of intestinal anastomoses and laparotomy wounds. *Acta Chir. Scand.*, **151**, 441–443.

KEMENY N, LOKICH JJ, ANDERSON N AND AHLGREN JD. (1993). Recent advances in the treatment of advanced colorectal cancer. *Cancer*, **71**, 9–18.

KIMBALL ES, SCHNEIDER CR, FISHER MC AND CLARK MC. (1992). Levamisole causes differential cytokine expression by elicited mouse peritoneal macrophages. *J. Leuk. Biol.*, **52**, 349–356.

MARTENS MFWC AND HENDRIKS T. (1991). Postoperative changes in collagen synthesis in intestinal anastomoses of the rat. Differences between small and large bowel. *Gut*, **32**, 1482–1487.

MARTENS MFWC, HENDRIKS T, WOBBES T AND DE PONT JJHHM. (1992*a*). Intraperitoneal cytostatics impair early post-operative collagen synthesis in experimental intestinal anastomoses. *Br. J. Cancer*, **65**, 649–654.

MARTENS MFWC, DE MAN BM, HENDRIKS T AND GORIS RJA. (1992*b*). Collagen synthesis throughout the uninjured and anastomosed intestinal wall. *Am. J. Surg.*, **164**, 354–360.

MAYER RJ. (1992). Chemotherapy for metastatic colorectal cancer. *Cancer*, **70**, 1414–1424.

MOERTEL C, FLEMING T, MACDONALD JS, HALLER D, LAURIE J, GOODMAN P, UNGERELEIDER J, EMMERSON W, TORMEY D, GLICK J, VEEDER M AND MAILLARD J. (1990). Levamisole and fluorouracil for adjuvant therapy of resected colon cancer. *N. Engl. J. Med.*, **322**, 399–401.

NORDLINGER B, PANIS Y, PUTS JP, HERVE JP, DELELO R AND BALLET F. (1991). Experimental model of colon cancer: recurrences after surgery alone or associated with intraperitoneal chemotherapy. *Dis. Colon Rectum*, **34**, 658–663.

PETERKOFSKY B, CHOIKIER M AND BATEMAN J. (1981). Determination of collagen synthesis in tissue and cell culture systems. In *Immunochemistry of the Extracellular Matrix*, Vol. 2, Furthmayer H. (ed.) pp. 19–47. CRC Press: Boca Raton, FL.

DE ROY VAN ZUIDEWIJN DBW , HENDRIKS T, WOBBES T AND DE BOER HHM. (1991). Intraperitoneal cytostatics impair healing of experimental intestinal anastomoses. *Br. J. Cancer*, **63**, 937–941.

SMITH PK, KROHN RI, HERMANSON GT, MALLIA AK, GARTNER FH, PROVENZANO MD, FUJIMOTO EK, GOEKE NM, OLSON BJ AND KLENK DC. (1985). Measurement of protein using bicinchoninic acid. *Anal Biochem*, **150**, 76–85.

STEVENSON HC, GREEN I, HAMILTON JM, CALABRO BA AND PARKINSON DR. (1991). Levamisole: known effects on the immune system, clinical results, and future applications to the treatment of cancer. *J. Clin. Oncol.*, **9**, 2052–2066.

SUGARBAKER PH. (1994). Intraperitoneal chemotherapy for treatment and prevention of peritoneal carcinomatosis and sarcomatosis. *Dis. Colon Rectum*, **37**, (suppl.), S115–S122.

SUGARBAKER PH, GRAVES T, DEBRUIJN EA, CUNLIFFE WJ, MULLINS RE, HULL WE, OLIFF L AND SCHLAG P. (1990). Early postoperative intraperitoneal chemotherapy as an adjuvant therapy for surgery for peritoneal carcinomatosis from gastrointestinal cancer: pharmacological studies. *Cancer Res.*, **50**, 5790–5794.

DE WAARD JWD, WOBBES TH AND HENDRIKS T. (1993). Early post-operative 5-fluorouracil does not affect the healing of experimental intestinal anastomoses. *Int. J. Colorect. Dis.*, **8**, 175–178.

DE WAARD JWD, WOBBES TH, VAN DER LINDEN CJ AND HENDRIKS T. (1995). Vitamin A may promote 5-fluorouracil-suppressed healing of experimental intestinal anastomoses. *Arch Surg.*, (in press).

WINDLE R, BELL PRF AND SHAW D. (1987). Five year results of a randomized trial of adjuvant 5-fluorouracil and levamisole in colorectal cancer. *Br. J. Surg.*, **74**, 569–572.